# Dietary Approaches to Stop Hypertension (DASH) diets and breast cancer among women: a case control study

**DOI:** 10.1186/s12885-020-07209-1

**Published:** 2020-07-29

**Authors:** Zeinab Heidari, Elahe Mohammadi, Vahideh Aghamohammadi, Saba Jalali, Arezoo Rezazadeh, Fatemeh Sedaghat, Mojan Assadi, Bahram Rashidkhani

**Affiliations:** 1 Department of Community Nutrition, Faculty of Nutrition Sciences and Food Technology, National Nutrition and Food Technology Research Institute, Shahid Beheshti University of Medical Sciences, Tehran, Iran; 2 Department of Nutrition, Khalkhal University of Medical Sciences, Khalkhal, Iran; 3 Department of Basic Medical Sciences, Faculty of Nutrition Sciences and Food Technology, National Nutrition and Food Technology Research Institute, ShahidBeheshti University of Medical Sciences, No 46, Hafezi Street, Farahzadi Boulevard, Sharak Ghods, P.O. Box: 1981619573, Tehran, Iran; 4 Department of Oncology, Shahid Madani Hospital, Alborz University of Medical Science, Karaj, Iran

**Keywords:** Breast cancer, Diet, DASH diet, Case-control

## Abstract

**Background:**

Studying entire dietary patterns is a promising alternative approach to overcome limitations of the single food or nutrient approach. We evaluated the relationship between the scores of 4 established Dietary Approaches to Stop Hypertension (DASH) diet indexes and breast cancer risk among Iranian women.

**Methods:**

This case-control study was carried out on 408 eligible women (136 cases and 272 hospital-based controls). A validated 168 item semi-quantitative food frequency questionnaire was used for assessing usual dietary intakes. DASH index scores were generated based on predefined algorithms for each of the 4 previously described indexes (Dixon’s, Mellen’s, Fung’s and Günther’s DASH diet index). Unconditional logistic regression analysis was performed to estimate odds ratio (OR) and 95% confidence intervals (CIs) for score categories or quintiles of DASH diet indexes and breast cancer risk in multivariate adjusted models.

**Results:**

Women in the highest categories of the Mellen’s and Günther’s scores had lower odds of breast cancer than those in the lowest quintiles (Mellen’s OR:0.50; 95% CI:0.62–0.97; P-trend:0.02; Günther’s OR:0.48; 95% CI:0.25–0.93; P-trend:0.05). However, no significant associations were found between Dixon’s and Fung’s DASH score and breast cancer risk. Modification by menopausal status revealed that breast cancer risk was only reduced in postmenopausal women with higher scores on Mellen’s index (OR:0.24; 95% CI:0.08–0.68; P-trend:0.04).

**Conclusion:**

A greater adherence to 2 of the 4 DASH indexes (Mellen’s and Günther’s indexes) was associated with decreased risk of breast cancer.

## Background

Breast cancer, the most prevalent cancer in women, is a major public health problem worldwide [1]. Breast cancer is a leading cause of death among female both in developed and developing countries [2]. In Iran, breast cancer ranks first among diagnosed malignancies in women, comprising 24.4% of all cancers with the age-standardized incidence rates of 23.1 per 100,000, and is the fifth most common causes of death due to cancers [3].

Among environmental risk factors of breast cancer, diet has been considered as an important modifiable exposure [4]. However, epidemiological studies have reported conflicting results regarding the association between food intake and breast cancer risk [5, 6]. On the other hand, most of these studies have traditionally focused on the effects of individual foods and nutrients on cancer risk [6–8]. Although, some potential biological mechanisms that underlie observed associations can be identified, “single nutrient” approach may not detect small effects of single dietary components and can be limited by the multicollinearity of dietary intake variables [5, 7, 8]. Therefore, studying entire dietary patterns is a promising alternative approach to overcome limitations of the single food or nutrient approach, account for the combined effects of and synergy between single dietary components [5, 7, 8] and provides useful information for suggesting guidelines and public health recommendations [7]. The Dietary Approaches to Stop Hypertension (DASH) which emphasizes fruits and vegetables, plant proteins, moderate amounts of low-fat dairy products, and low amounts of sweets and sodium, is a healthy eating pattern recommended for the general public by the United States Department of Agriculture [7, 9, 10]. Though this dietary approach was at first suggested to reduce hypertension and cardiovascular disease risk, several previous studies reported the inverse association between DASH diet score and colorectal cancer [7, 11, 12]. It seems that DASH diet might be effective for cancer prevention, especially because some of its characteristics, like high fruit and vegetable consumption and low meat intake, have been implicated in the etiology of cancer [7, 10]. While several prospective [13–17] and case-control [18–22] studies have considered exploratory dietary patterns and breast cancer risk, few studies have examined the associations between DASH scores and breast cancer [5, 10, 23]. A prospective cohort study showed that a high DASH score reduced the risk of estrogen receptor negative (ER-) breast cancer [10] and another case-control study indicated an inverse association between adherence to the DASH eating plan and odds of breast cancer in Iranian women [23]. On the other hand, the association between habitual intake of the DASH dietary pattern and breast cancer has not been adequately investigated in the Middle East, where the dietary intakes are greatly different from those in Western countries. Moreover, these limited studies have equivalently adopted the operationalized approach proposed by Fung et al. to calculate the DASH index [12]. Therefore, the purpose of the current study was to compare scores of 4 established DASH diet indexes [11, 24–26] and evaluate their relations to breast cancer risk among Iranian women.

## Methods

### Subjects

This hospital-based case-control study was carried out on women aged ≥30 years who were admitted to Shohadaye Tajrish and Imam Hossein hospitals in Tehran, Iran from September 2015 to February 2016. Only patients with histopathologically proven breast cancer (and no history of other cancers) were designated as breast cancer patients. Eligible cases were all incident cases of breast cancer in past 6 months who did not undergo any cancer treatments at the time of interview. Exclusion criteria for cases were history of hormone replacement therapy, being pregnant or lactating and having special dietary habits such as vegetarian.

The control group was then selected randomly among women referred to the same hospitals for a broad spectrum of non-neoplastic diseases not related to known or suspected risk factors for breast cancer and their eating habits. The exclusion criteria for controls were history of physician-diagnosed cancer in any site, HRT and benign breast disease, pregnancy or breast-feeding, and having special dietary habits. Two controls were enrolled for each case and matched for diagnosis hospital, menopausal status and age (±5 years). The participation rate was 95% for cases and 89% for control and 92% for all of them. Of the 408 eligible subjects participated in this study, a total of 401 subjects (134 cases and 267 controls) were included in the final analysis. Five controls and 2 cases were excluded from study because their daily energy intakes were either > 3 or < 3 SD from the mean. The ethics committee of the National Nutrition and Food Technology Research Institute of Shahid Beheshti University of Medical Science approved the study protocol and a written informed consent was obtained from all volunteers before enrolment in the study.

### Data collection

Questionnaire data regarding socio-demographic variables, history of cancer and other diseases, family history of cancer, reproductive history, HRT and vitamin D supplement use, and current or past smoking behavior were collected from participants at baseline. Information on the subject’s activity level was gathered using a valid physical activity questionnaire [27] and was then quantified in form of metabolic equivalent hour/day (METs-h/d). This method has been described in detail elsewhere [27, 28]. Weight was measured using digital scale (Seca, Germany) while the subjects were minimally clothed without shoes and recorded to the nearest 100 g. Height was measured via a wall mounted stadiometer (Seca, Germany) with 2 mm precision, while the participants wearing no shoes. The ratio of weight (in kg) to square of height (in meter) was used to determine the individual’s body mass index (BMI).

### Dietary assessment

We used a validated 168 item semi-quantitative food frequency questionnaire (FFQ) with multiple choice frequency response options for assessing usual dietary intakes of all participants. Reproducibility and relative validity of this FFQ in evaluating major dietary patterns and food and nutrient intake among Iranian adults have already been demonstrated [29]. Subjects were asked to provide the frequency of consumption of certain portions of each food on a daily, weekly, monthly or yearly basis throughout the preceding year before cancer diagnosis (for cases) or hospital admission (for controls). By using household measures, the portion sizes for each food item were converted to grams. Specified portion size, dish composition, and the average of reported frequency (e.g., divided by 30 if once a month) was taken into consideration to calculate daily value for each food item. To calculate the FFQ nutrient intakes, the modified Nutritionist IV software was used. The modification was done to include the Iranian foods in the original USDA food composition table embedded in the software.

### The DASH score

Computation of the DASH scores has previously been described in detail [7]. DASH index scores were generated based on the separate indexes defined by Mellen, Fung, Dixon, and Günther [11, 24–26]. Table 1 shows scoring standards and points for use in all these indexes. Dixonʾs DASH diet index includes 8 food groups and one nutrient (total fruits, total vegetables, whole grains, total dairy products, nuts/seeds/legumes, meat/meat equivalent, added sugar, saturated fat and alcohol). One point is assigned to each one. The total score is the sum of the individual 9 components scores, which ranges 0 to 9 scores. However, in our study alcohol components were removed from the Dixon’s DASH score due to religious practices. The recommended cut-off values for energy intakes were 1600 and 2000 kcal/d for women and men, respectively.

**Table 1:** Standards for maximum scores on 4 DASH diet indexes

Standards for maximum score				
	Dixon’s DASH index In women^a^ Sex-specific (women)	Mellen’s DASH index^b^	Fung’s DASH index^c^ Sex-specific (women)	Günther’s DASH index^d,e,^ Based on age, sex and activity
Dietary components for which greater intakes receive higher score				
Total fruit	≥4 servings/d^f^		Fifth quintile	≥4 servings/d^f^
Total vegetables	≥3 servings/d^f,g^			≥4 servings/d^f^
Vegetables without potatoes			Fifth quintile	
Total grains				≥6 servings/d^f^
Whole grains	≥4 servings/d^f,g^		Fifth quintile	
High-fiber grains				≥50% of total grain servings/d^f,h^
Total dairy products	≥2 servings/d^f^			≥2 servings/d^6^
Low-fat dairy products			Fifth quintile	≥75% of total dairy servings/d^f,h^
Nuts, seeds, legumes	≥3 servings/d^f^		Fifth quintile	≥4 servings/wk^f^
Protein		≥18% of total daily kcal		
Fiber		≥14.8 g/1000 kcal per day		
Magnesium		≥238 mg/1000 kcal per day		
Calcium		≥590 mg/1000 kcal per day		
Potassium		≥2238 mg/1000 kcal per day		
Dietary components for which lower intakes receive higher scores				
Meat/meat equivalents	< 6 oz. (170 g)/d^f^			
Meat, poultry, fish, eggs				≤2 servings/d^f^
Red and processed meat			First quintile	
Sugar-sweetened beverages			First quintile	
Sweets				≤5 servings/wk^f^
Fats, oils				≤3 servings/d^f^
Added sugar	≤3% of total daily kcal			
Total fat		27% of total daily kcal		
Saturated fat	≤5% of total daily kcal	≤6% of total daily kcal		
Cholesterol		≤71.4 mg/1000 kcal per day		
Sodium		≤1143 mg/1000 kcal per day	1st quintile	
Total score (points)	0–8	0–9	8–40	0–80

^a^Subjects receive 0 points for not meeting and 1 points for meeting the recommendation

^b^Subjects receive 1 points for meeting a target, 0.5 points for meeting an intermediate target, and 0 points for meeting neither target

^c^For recommended components, the highest quintile receives 5 points, and the lowest quintile receives 1 point; for components for which lower intakes are desirable, the lowest quintile of intake receives 5 points and the highest quintile of intake receives 1 point

^d^Standards are based on recommendations for a 2000 cal diet; different standards are available for 3 other energy intakes (1600, 2300, and 3100 kcal) according to sex, age and levels of physical activity

^e^Components are scored from 0 to 10, except for total dairy, low-fat dairy, whole grains, and high-fiber grains which are scored from 0 to 5

^f^Values are based on the Pyramid Servings database

^g^A total of 4 servings were based on the Dietary Guidelines recommendation for most grains to be whole, that Dixon et al. defined as 67% [11]

^h^If servings of total grains or total dairy were 0, components of high-fiber grains or low-fat dairy products would receive 0 points

Mellen et al. [26] designed a totally nutrient-based DASH diet index (Table 1). Greater adherence to the Mellen’s DASH diet index was associated with higher intakes of potassium, protein, fiber, magnesium, and calcium, and lower intakes of cholesterol, sodium, total fat, and saturated fat. The daily nutrient goal was set to a 2100 kcal/d diet, regardless of subject’s gender. One point assigned to those who meet the goal for each component, those who meet an intermediate goal receive 0.5 point, and 0 points was attributed to those who do not meet either of the two goals. The total score ranges from 0 to 9 [7, 26].

Fung’s index is the traditional DASH diet scoring system and includes 8 components highlighted or minimized in the DASH diet: high intakes of whole grains, fruits (includes fruit juice), vegetables (excludes potatoes), low-fat dairy products, and nuts and legumes, as well as low intakes red and processed meats, sweetened beverages, and sodium. The scoring system is based on quintile categories of eating the mentioned food items. For recommended components, those in the lowest quintile of intake receive 1 point and those in the highest quintile receive 5 points. In contrast, for components for which lower intakes are favorable, those in the highest and the lowest quintile of intake receives1 and 5 points, respectively. Component scores were then summed up to construct an overall adherence to DASH score, ranging from 8 to 40.

A more complex food-based DASH diet index has been defined by Günter et al. [25]. This index relies on 10 components to evaluate the individual’s compliance to the DASH diet plan. In accordance to their scales, these components are divided as follows:

- Six components on a 10-point scale, which include: (i) fruits and fruit juice, (ii) vegetables and potato, (iii) meat, poultry, fish and eggs, (iv) nuts, seeds and legumes, (v) fats and oils, and (vi) sweets.
- Four components with a 5-point scale, including: (i) total dairy, (ii) low-fat dairy, (iii) whole grains, and (iv) high-fiber grains.

Recommendations for 4 various energy intakes including 1600, 2000, 2300, and 3100 kcal/d are the basis of target intakes for each component that accounts for activity level, sex, and age defined by Dietary Reference Intakes (25). The ultimate DASH index is calculated by adding up the acquired points, and it yields a value in the range of 0 to 80.

### Statistical analysis

The statistical analyses were carried out in the SPSS commercial package, version 18 (SPSS Inc., Chicago, IL, USA). All the significance tests were performed with the confidence interval of, at least, 95% (corresponding to a *p*-value ≤0.05). In order to conduct statistical analyses on all the above-mentioned DASH scores (Mellen’s, Fung’s, and Günther’s) in this study, they were expressed as distribution-based indexes and the lowest quintile was considered as the referent category. Given the fact that Dixon’s DASH index is a whole numbers 9-point scale with a limited range of values, score categories ≤1 (referent category), 2, 3, and ≥ 4 were selected. Unconditional logistic regression analysis was performed to estimate odds ratio (OR) and their corresponding 95% confidence intervals for score categories or quintiles of DASH diet indexes and breast cancer risk in multivariate adjusted models. The possibilities of effect modification by menopausal status were considered by additional models. Moreover, complementary analyses were performed to examine whether individual components of DASH index are independently associated with the risk of breast cancer. All multivariable models were adjusted for the following covariates: age (y), BMI (in kg/m^2^), physical activity, smoking, total energy intake (kcal/d), vitamin D supplement use, age at first live birth, and family history of cancer. Furthermore, in order to compare total scores on the 4 indexes, Spearman’s correlation coefficients were calculated.

## Results

Table 2 shows the baseline characteristics according to categories or quintiles of total DASH scores for all indexes. Women in control group with high scores on Mellen’s and Dixon’s indexes tended to start their menarche at a slightly older age. Also, women in control group with high scores on Mellen’s index were older. In both case and control groups, women with higher scores on all indexes had higher energy intake, the only exception was with the Mellen’s index which is an energy adjusted model. Women in control group with high scores on Fung’s index were more physically active.

**Table 2:** Characteristics of subjects according to category or quintiles of 4 DASH diet index scores

	Dixon’s DASH index^a^			Mellen’s DASH index			Fung’s DASH index			Günther’sDASH index		
	< 1 point	≥4 points	*P*-value	Quintile 1	Quintile 5	*P*-value	Quintile 1	Quintile 5	*P*-value	Quintile 1	Quintile 5	*P*-value
Median score												
Cases	1	2		1	4		17	31		53	70	
Control	1	2		1	4		19	31		54	70	
Number												
Cases	61	17		39	24		44	26		41	30	
Control	89	38		51	56		53	46		53	53	
Age												
Case	48.0 ± 10.0^b^	51.0 ± 10.0	0.38	48.0 ± 11.0	49.0 ± 10.0	0.31	47.0 ± 10.0	50.0 ± 9.0	0.20	46.0 ± 10.0	50.0 ± 9.0	0.20
Control	47.0 ± 10.0	49.0 ± 10.0	0.20	43.0 ± 7.0	49.0 ± 11.0	0.03	47.0 ± 9.0	51.0 ± 8.0	0.01	49.0 ± 10.0	45.0 ± 12.0	0.25
Weight												
Case	73.0 (20. 0)^c^	74.0 (15.0)	0.82	75.0 (22.0)	72.0 (18.0)	0.92	73.0 (16.0)	75.00 (14.0)	0.25	76.0 (26.0)	72.0 (22.0)	0.82
control	72.0 (20.0)	68.0 (20.0)	0.56	68.0 (12.0)	75.0 (19.0)	0.03	72.0 (13.0)	71.00 (10.0)	0.99	72.0 (19.0)	70.00 (23.0)	0.47
Height												
Case	158.0 (7.0)^c^	160.0 (4.0)	0.32	158.0 (10)	155.0 (9.0)	0.43	158.0 (4.0)	157.0 (5.0)	0.58	159.0 (7.0)	157.0 (9.0)	0.13
Control	159.0 (8.0)	158.0 (5.0)	0.72	157.0 (6.0)	160.0 (9.0)	0.14	159.0 (5.0)	158.0 (5.0)	0.45	158.0 (8.0)	158.0 (28.0)	0.94
BMI												
Case	28.0 (8.0)^c^	28.0 (17.0)	0.31	29.0 (7.0)	29.0 (6.0)	0.99	29.0 (6.0)	30.0 (5.0)	0.12	28.0 (8.0)	29.0 (7.0)	0.56
Control	28.0 (6.0)	27.0 (6.0)	0.64	27.0 (4.0)	29.0 (6.0)	0.34	28.0 (5.0)	28.0 (4.0)	0.96	28.0 (6.0)	27.0 (9.0)	0.73
Energy intake												
Case	2167.0 (635.0)^c^	3210.0 (470.0)	< 0.001	2558.0 (921.0)	2764.0 (925.09)	0.17	2200.0 (614.0)	2914.0 (951.0)	< 0.001	2012.0 (320.0)	2778.0 (803.0)	< 0.001
Control	2177.0 (631.0)	3411.0 (1412.0)	< 0.001	2541.0 (1021.0)	2622.0 (1069.0)	0.95	2355.0 (784.0)	3307.0 (1245.0)	< 0.001	1962.0 (377.0)	3199.0 (1616.0)	< 0.001
Physical activity score												
Case	31.0 (5.0)^c^	33.0 (8.0)	0.13	32.0 (5.0)	33.0 (6.0)	0.10	30.0 (7.0)	33.0 (6.0)	0.08	32.0 (7.0)	32.0 (5.0)	0.95
Control	31.0 (5.0)	32.0 (7.0)	0.86	31.0 (5.0)	33.0 (6.0)	0.26	31.0 95.0)	33.0 (6.0)	0.03	32.0 (5.0)	32.0 (5.0)	0.59
Menarche age												
Case	14.0 (1.0)^c^	14.0 (2.0)	0.58	13.0 (2.0)	13.0 (2.0)	0.39	14.0 (1.0)	13.0 (2.0)	0.74	14.0 (2.0)	14.0 (2.0)	0.71
Control	13.0 (1.0)	14.0 (2.0)	0.02	13.0 (1.0)	14.0 (2.0)	0.02	13.0 (2.0)	14.0 (2.0)	0.17	13.0 (1.0)	14.0 (1.0)	0.82
Menopause status												
Case status			0.004			< 0.001			< 0.001			
Premenopause	29 (47.5)^d^	6 (35)		19 (49)	13 (54)		21 (48)	11 (42)		9 (46)	15 (50)	< 0.001
Postmenopause	32 (52.5)	11 (65)		20 (51)	11 (36)		23 (52)	15 (58)		22 (54)	15 (50)	
Control status			0.06			< 0.001			0.01			< 0.001
Premenopause	52 (58)	19 (50)		34 (68)	30 (53.5)		28 (52)	17 (37)		27 (51)	30 (56)	
Postmenopause	38 (42)	19 (50)		16 (32)	26 (46.5)		25 (48)	29 (63)		26 (49)	23 (44)	

^a^Dixon’s DASH index scores were grouped into 4 categories [≤1 (*n* = 151), 2 (*n* = 101), 3 (*n* = 94), and ≥ 4 (*n* = 55) points] because of a limited range of values. DASH, Dietary Approaches to Stop Hypertension

^b^Mean ± SE (all such values)

^c^Median (IQR) (all such values)

^d^Number (Percent) (all such values)

Table 3 presents the correlations between total scores for all DASH indexes. Correlation coefficients ranged from 0.07 to0.69.The highest correlation was observed between Fung’s and Dixon’s indexes(*r* = 0.69), while the weakest correlation was between Gunther’s and Mellen’s DASH indexes (*r* = 0.07).

**Table 3:** Spearman’s correlation coefficients in summary scores for 4 DASH diet indexes^a^

	Dixon’s DASH index	Mellen’s DASH index	Fung’s DASH index	Günther’s DASH index
Dixon’s DASH index	1.00	0.27	0.69	0.58
Mellen’s DASH index	0.27	1.00	0.29	0.07
Fung’s DASH index	0.69	0.29	1.00	0.61
Günther’s DASH index	0.58	0.07	0.61	1.00

^a^*P*<0.0001

The ORs and their 95% CI for total DASH scores and breast cancer risk are shown in Table 4. After controlling for potential confounders, women in the highest quintiles of the Mellen’s and Günther’s scores had 50, and 52% lower odds of breast cancer than those in the lowest quintiles respectively (Mellen’s OR: 0.50; 95% CI: 0.62–0.97; P-trend: 0.02;Günther’s OR: 0.48; 95% CI: 0.25–0.93, P-trend: 0.05). However, no significant associations were found between Dixon’s and Fung’s DASH score and breast cancer risk.

**Table 4:** Multivariable adjusted ORs^a^ (95% CIs) for breast cancer in women by category or quintiles of DASH diet index scores

DASH index	Quintiles of DASH index					P- trend
	Q1	Q2	Q3	Q4	Q5	
**Dixon’s DASH index**						
All women						
Cases/controls	61 / 90	29 / 72	27 / 66	17 / 38	–	
Crude OR	1.00 (referent)	0.59 (0.34–1.02)	0.59 (0.34–1.03)	0.66 (0.34–1.27)		0.08
Multivariate OR	1.00 (referent)	0.79 (0.42–1.45)	0.80 (0.43–1.52)	0.94 (0.42–2.13)	–	0.71
Premenopause						
Case/control	29 / 52	14 / 43	13/ 38	6 / 19	–	
Multivariate OR	1.00 (referent)	0.81 (0.33–1.97)	0.85 (0.35–2.04)	0.91 (0.28–2.96)	–	0.79
Postmenopause						
Cases/controls	32 / 38	15 / 29	14 / 28	11 / 19	–	
Multivariate OR	1.00 (referent)	0.67 (0.27–1.66)	0.56 (0.21–1.54)	0.70 (0.21–2.29)	–	0.39
**Mellen’s DASH index**						
All women						
Cases/controls	39 / 50	38 / 75	19 / 47	14 / 38	24 / 56	
Crude OR	1.00 (referent)	0.66 (0.37–1.17)	0.52 (0.26–1.04)	0.48 (0.23–1.01)	0.56 90.29–1.05)	0.04
Multivariate OR	1.00 (referent)	0.61 (0.34–1.09)	0.48 (0.24–0.96)	0.43 (0.20–0.92)	0.50 (0.62–0.97)	0.02
Premenopause						
Cases/controls	19 / 34	18 / 36	8 / 29	4 / 23	13 / 30	
Multivariate OR	1.00 (referent)	0.84 (0.37–1.91)	0.48 (0.18–1.26)	0.30 (0.09–1.02)	0.78 (0.32–1.87)	0.22
Postmenopause						
Cases/controls	20/ 16	20 / 39	11 / 18	10 / 15	11 / 26	
Multivariate OR	1.00 (referent)	0.26 (0.10–0.67)	0.30 (0.10–0.67)	0.34 (0.11–1.05)	0.24 (0.08–0.68)	0.04
**Fung’s DASH index**						
All women						
Cases/controls	44 / 53	19 / 70	17 / 53	28 / 45	26 / 46	
Crude OR	1.00 (referent)	0.32 (0.17–0.62)	0.38 (0.19–0.76)	0.74 (0.40–1.39)	0.68 (0.36–1.27)	0.71
Multivariate OR	1.00 (referent)	0.31 (0.16–0.62)	0.40 (0.19–0.83)	0.74 (0.38–1.44)	0.49 (0.25–0.98)	0.28
Premenopause						
Cases/controls	21 / 28	9 / 44	7 / 36	14 / 27	11 / 17	
Multivariate OR	1.00 (referent)	0.27 (0.11–0.69)	0.25 (0.08–0.72)	0.68 (0.27–1.67)	0.74 (0.27–2.03)	0.73
Postmenopause						
Cases/controls	23 / 25	10 / 26	10 / 16	14 / 18	15 / 29	
Multivariate OR	1.00 (referent)	0.38 (0.13–1.05)	0.62 (0.21–1.81)	0.50 (0.17–1.41)	0.36 (0.13–0.94)	0.07
**Günther’s DASH index**						
All women						
Cases/controls	41 / 53	25 / 53	17 / 54	21 / 53	30 / 53	
Crude OR	1.00 (referent)	0.51 (0.27–0.97)	0.26 (0.12–0.56)	0.86 (0.48–1.54)	0.52 (0.27–0.99)	0.22
Multivariate OR	1.00 (referent)	0.41 (0.21–0.82)	0.36 (0.17–0.75)	0.48 (0.24–0.95)	0.48 (0.25–0.93)	0.05
Premenopause						
Cases/controls	19 / 27	13 / 31	8 / 36	7 / 28	15 / 30	
Multivariate OR	1.00 (referent)	0.33 (1.25–0.91)	0.26 (0.09–0.79)	0.37 (0.12–1.12)	0.57 (0.22–1.5)	0.37
Postmenopause						
Cases/controls	22 / 26	12 / 22	9 / 18	14 / 25	15 / 23	
Multivariate OR	1.00 (referent)	0.42 (0.15–1.16)	0.43 (0.14–1.29)	0.52 (0.19–1.37)	0.50 (0.18–1.34)	0.21

Dixon’s DASH index scores were grouped into 4 categories (≤1, 2, 3, and ≥ 4 points) because of a limited range of values (total score range is 0–9).OR: odds ratio, 95% CI: 95% confidence interval. Statistically significant *P*-values are reported in bold, *p ≤ 0.05 considered as significant

^a^Adjusted for age, BMI, energy intake, physical activity, age at first live birth, vitamin D supplements and family history of cancer

Further stratification by menopausal status revealed that Dixon’s DASH diet index, Fung’s DASH diet index, and Günther’s DASH diet index were not significantly associated with breast cancer risk between premenopausal and postmenopausal women. However, risk estimates were significantly reduced for breast cancer in postmenopausal women with higher scores on Mellen’s DASH index (Multivariate OR: 0.24; 95% CI: 0.08–0.68; P-trend: 0.04).

Table 5 shows the multivariate adjusted ORs and 95% Confidence Intervals (CIs) for breast cancer risk by-component analyses (mutual adjustment). Each individual component was examined solely, with the total index score minus the relevant component controlled for. Among components in which higher intakes captured greater scores, the following were significantly related to reduced risk of breast cancer: total fruits, total grains and total dairy product with Dixon’s DASH index, potassium with Mellen’s DASH index, total fruit, vegetables (without potatoes), and low-fat dairy products with Fung’s DASH index, and total vegetables, total dairy products, and low-fat dairy products with the Günther’s DASH index.

**Table 5:** Multivariable-adjusted ORs (95% CIs) for breast cancer for highest compared with lowest categories or quintiles of DASH individual component scores for each index in women^a^

	Dixon’s DASH index^**b**^	Mellen’s DASH index^**b**^	Fung’s DASH index^**c**^	Günther’s DASH index^**c**^
Dietary components for which greater intakes receive higher score				
Total fruit	0.68 (0.44–1.04)	–	0.42 (0.21–0.84)	0.95 (0.84–1.08)
Total vegetables	0.50 (0.33–0.77)	–	–	0.87 (80–0.96)
Vegetables without potatoes	–	–	0.49 (0.25–0.98)	–
Total grains	–	–	–	1.03 (0.81–1.31)
Whole grains	0.51 (0.36–0.86)	–	1.83 (0.89–3.73)	–
High-fiber grains	–	–	–	1.15 (0.73–1.82)
Total dairy products	0.54 (0.35–0.83)	–	–	0.83 (0.70–0.99)
Low-fat dairy products	–	–	0.46 (0.23–0.92)	0.79 (0.63–0.99)
Nuts, seeds, legumes	1.36 (0.84–2.19)	–	0.96 (0.46–2.00)	1.04 (0.94–1.51)
Protein	–	1.49 (0.50–4.39)	–	–
Fiber	–	0.69 (0.34–1.38)	–	–
Magnesium	–	0.83 (0.52–1.32)	–	–
Calcium	–	0.80 (0.50–1.29)	–	–
Potassium	–	0.61 (0.40–0.94)	–	–
Dietary components for which lower intakes receive higher scores				
Meat/meat equivalents	1.10 (0.37–3.25)	–	–	–
Meat, poultry, fish, eggs	–	–	–	0.96 (0.82–1.14)
Red and processed meat	–	–	2.03 (1.00–4.14)	–
Sugar-sweetened beverages	–	–	0.75 (0.42–1.34)	–
Sweets	–	–	–	1.01 (096–1.07)
Added sugar	0.88 (0.57–1.36)	–	–	–
Fats, oils	–	–	–	1.03 (098–1.05)
Total fat	–	0.94 (0.59–1.51)	–	–
Saturated fat	1.01 (0.99–1.06)	071 (0.46–1.08)	–	–
Cholesterol	–	0.87 (0.55–1.37)	–	–
Sodium	–	1.16)0.63–2.12)	1.49 (0.76–2.92)	–

^a^Multivariable adjusted ORs (95% CIs) were conducted for each component with adjustment for the total DASH score without the respective component in addition to age, BMI, energy intake, physical activity, age at first live birth, vitamin D supplements and family history of cancer

^b^Meeting the recommendation (1 point) compared with not meeting the recommendation (0 points)

^c^Quintile 5 compared with quintile

## Discussion

To the best of our knowledge, our study is the first observational study that epidemiologically examined the association between 4 DASH diet indexes and breast cancer risk. In present case-control study, we found that a greater adherence to 2 of the 4 DASH indexes (Mellen’s and Günther’s indexes) was associated with markedly decreased risk of breast cancer in Iranian women. After stratified by menopausal status, only Mellen’s DASH index was linked to reduced risk of breast cancer, among postmenopausal women. The available evidence linking the DASH diet to breast cancer is limited. Fung et al. reported that the whole DASH was significantly associated with a lower risk of ER-breast cancer in postmenopausal women [10]. Recently, Soltani et al. also revealed that in Iranian women the highest quartile of the DASH diet score had 85% lower odds of breast cancer than women in the bottom quartile. Stratified analysis showed such association in postmenopausal women, but not in premenopausal women [23].

The results of current study suggest that the main effective components of the DASH diet, which can protect against breast cancer, may exist in almost all indexes. On the other hand, the lack of an association between Dixon’s and Fung’s DASH scores and breast cancer in our research demonstrates that differing in composition of the indexes (some indexes emphasize on food choices; however, the other may be nutrient-based or calorie-based) and the scoring procedures may affect the results. Dixon and Günther indexes use certain cutoffs and Fung index applies rankings of intakes whereas Mellen’s index includes a density-based method (ie, intakes are evaluated relative to total calorie). Furthermore, Dixonʾs and Mellen’s indexes directly assess the saturated fat intake while both indexes developed by Fung and Günther estimate the intake of saturated fat indirectly through consumption of saturated fat rich foods [11, 24–26]. Finally, it is important to understand the inherent difference of DASH index formulation. In Dixon’s method, meat and meat equivalent consumption have been considered. However, Fung’s index notices intake of red and processed meat, and in Günther’s index intake of meat, poultry, fish, and eggs is important [11, 24, 25].

In a cohort study by Miller et al., men with the highest scores on all 4 of the indexes and women with the highest scores on Mellen’s, Fung’s, and Günther’s indexes had significant reduced risk of colorectal cancer [7]. The Fung’s index consists of 8 components including 7 food groups and one nutrient (sodium) [10]. This index has been used frequently in studies which investigated the association between DASH dietary pattern and diseases [10, 30]. Unlike our results, Hirko et al. reported that a significant reduced risk of human epidermal growth factor 2 positive breast cancer was observed among women in 5th vs. 1st quintile of DASH scores based on Fung’s index [30]. However, Fung’s score had no significant association with other molecular subtypes of breast cancer in this study [30]. Similar significant inverse association between a high DASH score based on Fung’s index and ER-breast cancer but not of ER+ breast cancer, was reported in a study by Fung et al. [10].

In previous studies on dietary patterns and breast cancer risk in Iranian women, a ‘healthy’ dietary pattern characterized by the consumption of vegetables, fruits, low-fat dairy products, legumes, olive and vegetable oils, fish, condiments, organ meat, poultry, soya and whole grains was inversely related to breast cancer risk [21]. Moreover, the results of another study by Heidari et al. showed significant increase in breast cancer risk in women in the highest category of the unhealthy dietary pattern (OR: 2.21; 95%CI: 1.04, 4.690; P-trend = 0.009) [30]. The mentioned study also showed that after stratifying by menopausal status, the association between breast cancer risk and unhealthy dietary pattern was statistically significant only among post-menopausal women (OR: 3.56; 95%CI: 1.16, 10.95; P-trend = 0.008) [28]. In our study, component analysis revealed that in overall, greater intake of fruits, total vegetables, vegetables without potatoes, grains, total dairy product, low fat dairy products and potassium was significantly associated with reduced risk of breast cancer. Fruits and vegetables are rich sources of antioxidants, vitamins, minerals and fiber which have been shown to play a protective role against breast cancer [10, 31].

Comparing 4 DASH diet indexes in the same study for the same outcome is the main strength of our study. In addition, only recently diagnosed women with breast cancer (within the past 6 months) were enrolled in this study. Therefore, alteration in the diet by cases due to cancer diagnosis is less possible. The high participation rate of the study subjects and adjusting the analysis for confounding variables were the other strength of the study. Where economic resources are severely limited, food consumption is strongly correlated to income so that even little income differences are directly reflected in diet. Thus, studies in developing countries can offer unique opportunities to investigate the association between diet and cancer (due to large between person variation) [32]. Moreover, dietary patterns are likely to vary according to geographic region, socio-economic status, cultural practices, and food preferences and availability [28]. Especially, dietary intake of the Middle-Eastern population has its own unique characteristics, being recognized by higher consumption of refined grains (white rice and bread) and hydrogenated fats and a higher percentage of energy from carbohydrates [28].

On the other hand, our study has some limitations. First, recall bias is possible due to the hospital-based case-control design of study. In case-control studies, cases may recall their previous diet differently in the context of their cancer diagnosis and this can affect the associations (overestimation). Second, the possibility of selection bias cannot be avoided in retrospective case-control studies. In the present study, the probability of selection bias was minimized by high participation rates and by selecting hospital controls from patients whose admission diagnosis was unrelated to alcohol intake, tobacco smoking, and diet-related diseases (limiting share exposure). Lack of precision of results due to small sample size is another limitation of our study. Moreover, although we used a validated food-frequency questionnaire for assessing the dietary intake, measurement errors that might led to underestimation or even over estimation of associations were inevitable [29]. Finally, breast cancer is a hormone-sensitive cancer and evidence indicates that dietary pattern may have an impact on some subtypes of breast cancer and no effect on others. For example, two prospective cohort studies showed that DASH scores were inversely associated with ER- breast cancer [5, 10]. Therefore, it was better to stratify the results by hormone receptor status. However, we didn’t collect the information about the cancer subtype of our patients. Additional well-designed studies without these limitations are needed to elucidate the role of various aspects of 4 DASH diet indexes in prediction of breast cancer risk and find a standardized DASH diet index in these patients.

## Conclusion

Overall, we demonstrated that 2 of 4 DASH diet indexes (Mellen’s and Günther’s) reduced breast cancer risk in women. Nevertheless, minor differences in scoring method of these indexes can affect the results which should be taken into consideration in future research.

## Data Availability

The data and materials are available from the corresponding author on reasonable request.

## Abbreviations

BMI: Body mass index
CIs: Confidence Intervals
DASH: Dietary Approaches to Stop Hypertension
ER: Estrogen receptor
FFQ: Food frequency questionnaire
HRT: Hormone replacement therapy
OR: Odds ratio
